# A rapid NGS strategy for comprehensive molecular diagnosis of Birt-Hogg-Dubé syndrome in patients with primary spontaneous pneumothorax

**DOI:** 10.1186/s12931-016-0377-9

**Published:** 2016-05-27

**Authors:** Xinxin Zhang, Dehua Ma, Wei Zou, Yibing Ding, Chengchu Zhu, Haiyan Min, Bin Zhang, Wei Wang, Baofu Chen, Minhua Ye, Minghui Cai, Yanqing Pan, Lei Cao, Yueming Wan, Yu Jin, Qian Gao, Long Yi

**Affiliations:** Jiangsu Key Laboratory for Molecular Medicine, Nanjing University Medical School, Nanjing, People’s Republic of China; Department of Cardiothoracic Surgery, Taizhou Hospital of Zhejiang Province, Wenzhou Medical University, Linhai, People’s Republic of China; Department of Thoracic Surgery, Nanjing Chest Hospital, Nanjing, People’s Republic of China; Nanjing Children’s Hospital, Nanjing, People’s Republic of China; Center for Translational Medicine, Nanjing University Medical School, 22 Hankou Rd, Nanjing, 210093 People’s Republic of China

**Keywords:** Primary spontaneous pneumothorax, BHD syndrome, FLCN, Targeted next generation sequencing, CNV analysis

## Abstract

**Background:**

Primary spontaneous pneumothorax (PSP) or pulmonary cysts is one of the manifestations of Birt-Hogg-Dube syndrome (BHDS) that is caused by heterozygous mutations in *FLCN* gene. Most of the mutations are SNVs and small indels, and there are also approximately 10 % large intragenic deletions and duplications of the mutations. These molecular findings are generally obtained by disparate methods including Sanger sequencing and Multiple Ligation-dependent Probe Amplification in the clinical laboratory. In addition, as a genetically heterogeneous disorder, PSP may be caused by mutations in multiple genes include *FBN1*, *COL3A1*, *CBS*, *SERPINA1* and *TSC1*/*TSC2* genes. For differential diagnosis, these genes should also be screened which makes the diagnostic procedure more time-consuming and labor-intensive.

**Methods:**

Forty PSP patients were divided into 2 groups. Nineteen patients with different pathogenic mutations of *FLCN* previously identified by conventional Sanger sequencing and MLPA were included in test group, 21 random PSP patients without any genetic screening were included in blinded sample group. 7 PSP genes including *FLCN, FBN1*, *COL3A1*, *CBS*, *SERPINA1* and *TSC1*/*TSC2* were designed and enriched by Haloplex system, sequenced on a Miseq platform and analyzed in the 40 patients to evaluate the performance of the targeted-NGS method.

**Results:**

We demonstrated that the full spectrum of genes associated with pneumothorax including *FLCN* gene mutations can be identified simultaneously in multiplexed sequence data. Noteworthy, by our in-house copy number analysis of the sequence data, we could not only detect intragenic deletions, but also determine approximate deletion junctions simultaneously.

**Conclusions:**

NGS based Haloplex target enrichment technology is proved to be a rapid and cost-effective screening strategy for the comprehensive molecular diagnosis of BHDS in PSP patients, as it can replace Sanger sequencing and MLPA by simultaneously detecting exonic and intronic SNVs, small indels, large intragenic deletions and determining deletion junctions in PSP-related genes.

## Background

Primary spontaneous pneumothorax (PSP, OMIM #173600) is defined as the spontaneous occurrence of pneumothorax in a healthy individual without obvious underlying lung disease. A positive family history is found in 11.5 % of PSP patients [1]. While some cases can be attributed to several rare inherited monogenic disorders caused by mutations in *FBN1*, *COL3A1*, *CBS*, *SERPINA1* and *TSC1*/*TSC2* genes [2–6], a significant proportion of PSP cases are caused by germline mutations of *FLCN* gene, a causative gene of Birt-Hogg-Dube syndrome (BHD, OMIM #135150) [7–11].

BHD syndrome is an autosomal dominant disease characterized by skin fibrofolliculomas, renal cancer, pulmonary cysts and spontaneous pneumothorax [12–15]. Since the clinical manifestations may be atypical and variable [16, 17], BHD patients could exhibit a pneumothorax-dominant phenotype with no or reduced penetrance of skin or renal manifestations, the syndrome is considered to be under-diagnosed [18–23]. *FLCN* mutation carriers have been reported to have an increased lifetime risk of developing renal cell carcinoma (RCC) [24, 25], they should be advised to take some preventive measures, largely aimed at early recognition and treatment of RCC. Under the circumstances, it is an imperative diagnostic approach to perform genetic testing in PSP patients [17].

Thus far, the molecular diagnosis of BHD syndrome is mainly based on Sanger sequencing. However, there are different types of mutations in *FLCN* gene, comprised of base pair substitutions, small indels, deletions and duplications [26–28], which make the diagnostic procedure complicated and inefficient. Moreover, for differential diagnosis, patients with PSP should also be screened for mutations in *FBN1*, *COL3A1*, *CBS*, *SERPINA1* and *TSC1*/*TSC2* genes. Together, this requires about 220 primer reactions in Sanger sequencing, plus at least 7 probe sets in Multiple Ligation-dependent Probe Amplification (MLPA) analysis.

Given the complicity, it is not feasible to conduct a comprehensive mutation screening in clinical diagnostics in PSP patients. In recent years, next generation sequencing (NGS) and target enrichment techniques have been further developed, allowing us to focus specifically on genomic regions of interest for cheaper multiplexed sequencing of more cases. It has been successfully used in diagnosis of genetically heterogeneous diseases, such as breast cancer and congenital muscular dystrophy [29, 30].

Herein we sought to develop a NGS-based method using Haloplex target enrichment and the MiSeq platform to identify not only point mutations and indels but also copy number variations (CNV) as an accurate and easier diagnostic tool for simultaneous mutation screening of all the PSP-related genes. Using little input DNA, this system promises a quicker, more affordable and efficient analysis in diagnostic laboratories. To this end, we used Haloplex to screen 7 genes responsible for PSP in 40 PSP patients and aimed to evaluate its performance in identifying SNPs, indels and copy number variations affecting target genes.

## Methods

### Patients

Forty unrelated patients with PSP who had given written informed consent participated in the genetic screen and were divided into two groups.

#### Test group

Nineteen patients with different pathogenic mutations of *FLCN* previously identified by conventional Sanger sequencing and MLPA were included in test group (patients F01 to F19) [7, 8].

#### Blinded sample group

Twenty-one random PSP patients without any genetic screening were included in blinded sample group (patients F20 to F40).

Subjects were recruited from two tertiary hospitals, Taizhou Hospital of Zhejiang Province and Nanjing Chest Hospital. All participants in this study completed a medical questionnaire. The presence of lung cysts was evaluated by chest computed tomography. Medical and surgical records were reviewed if available. Peripheral blood samples were collected for DNA extraction and subsequent analysis.

### Statement of ethical approval

The study was approved by the Ethical Committees of Taizhou Hospital of Zhejiang Province (TZYY-2011106) and the Ethical Committees of Nanjing Chest Hospital (NCH-2011062). Informed consent was obtained from all individual participants included in the study.

### DNA extraction, quantification, and quality control

Genomic DNA was extracted from peripheral blood samples of collected family members and controls using a Qiagen DNA Mini blood Kit (Qiagen, Hilden, Germany) according to manufacturer instruction. DNA purity and concentration were assessed by the NanoDrop2000 spectrophotometer (Thermo Fisher Scientific, Florida, USA), A260/A280 ratios of 1.8 to 2.0 were accepted. DNA fragmentation was assessed by agarose gel electrophoresis.

### HaloPlex design, target enrichment, and next generation sequencing

A custom Haloplex panel was designed using Agilent’s online SureDesign tool (https://earray.chem.agilent.com/suredesign/index.htm). In all, 78 target regions including the whole gene sequences of *FLCN*, *TSC1*, *TSC2*, *CBS*, *COL3A1* and all coding exons, intron-exon boundaries including 50 intronic nucleotides and 5′ UTR (Untranslated Regions), 3′ UTR of *FBN1*, *SERPINA1* were captured for enrichment (Table 1). According to previous reports, intragenic large deletions have been identified in *FLCN*, *TSC1*, *TSC2*, *CBS* and *COL3A1* genes [8, 31–33], most breakpoints of the deletions locate in intronic sequences. In order to assess the performance of breakpoint analysis using NGS system, we captured both exonic and intronic sequences. However, large deletions in *SERPINA1* gene were rarely reported. Blyth et al. (2008) suggested that Marfan patients with large deletions in *FBN1* gene have a more severe phenotype, and barely manifest as isolated pneumothorax [34]. Therefore, we only targeted coding exons, intron-exon boundaries, 5′- and 3′- UTRs of *SERPINA1* and *FBN1*genes. The predicted target coverage was 98.72 %. The 214 kb target regions were captured using the Agilent HaloPlex Target Enrichment System Kits for Illumina Sequencing (Custom Panel Tier 1, ILM, 48 reactions; Agilent Technologies, Inc. Santa Clare, CA) following Agilent protocols. In brief, 225 ng of genomic DNA was fragmented using eight different restriction enzymes and denatured. Hybridization was performed with a biotinylated custom probe library and sample indexes, and then the target DNA fragments were captured with magnetic streptavidin beads and circularized by ligation. The captured target libraries were amplified by PCR, quality controlled and quantified using the BioAnalyzer 2100 (Agilent Technologies, Inc. Santa Clare, CA). Equimolar amounts of differentially indexed samples were pooled before pair-ended sequencing 300 bp on the Illumina MiSeq platform (Illumina Inc., San Diego, CA, USA) and the mean read depth within the regions of interest was ~300 reads per base.

**Table 1:** Genomic regions targeted for PSP genes

Disease	Gene name	Chromosome location	Capture region	Size (bp)	Exon	Transcript
Birt–Hogg–Dube syndrome	FLCN	chr17:17115527–17145502	Whole gene	29976	14	NM_144997
Lymphangioleiomyomatosis	TSC1	chr9:135766735–135822020	Whole gene	55286	23	NM_000368
	TSC2	chr16:2095990–2138713	Whole gene	42724	42	NM_000548
Homocystinuria	CBS	chr21:44473301–44497053	Whole gene	23853	17	NM_000071
Ehlers-Danlos syndrome	COL3A1	chr2:189839046–189877472	Whole gene	38527	51	NM_000090
Marfan syndrome	FBN1	chr15:48700503–48938046	Exons	18559	66	NM_000138
α1- Antitrypsin deficiency	SERPINA1	chr14:94843084–94857030	Exons	4863	7	NM_001002236
			Capture size	213788	220	

### Bioinformatics analysis for NGS results

The authors implicated in the bioinformatics analysis had no information on the patient data. Post-run sequencing quality was assessed by FastQC (Babraham Bioinformatics, Cambridge, UK). Sequence reads were aligned to the hg19 human reference genome (February 2009 assembly), and analyzed with Agilent software Surecall v2.1.1.13 using the default Haloplex parameters. The mutation reports (*.vcf files) were then annotated using ANNOVAR [35]. After removing all SNVs/indels occurring within non-coding regions, the variations were filtered against dbSNP137 NonFlagged, 1000 genomes and ESP 6500 databases. Rare, non-synonymous, exonic variants were subjected to bioinformatics tool PolyPhen [36] and SIFT [37] to evaluate their possible impact on protein structure and function. To perform extensive analysis, we further analyzed the intron sequences to identify potential splice and branch-site mutations using splice prediction tool ESEfinder [38]. If any potentially harmful mutation identified, validation was performed by Sanger sequencing.

### Copy-number analysis

CNVs were identified using a depth-based method. Coverage statistics were generated using GATK Depth of Coverage tool version 3.1 [39, 40]. We normalized coverage in each sample by dividing the average coverage of each exon by the total number of on-target mapped reads for that sample $\left(\mathrm{normalized},\mathrm{coverage},=,\frac{\mathrm{the}\ \mathrm{average}\ \mathrm{coverage}\ \mathrm{of}\ \mathrm{each}\ \mathrm{exon}}{\mathrm{the}\kern0.22em \mathrm{total}\;\mathrm{number}\;\mathrm{of}\;\mathrm{on}-\mathrm{target}\;\mathrm{mapped}\;\mathrm{reads}\;for\;\mathrm{that}\;\mathrm{sample}}\right)$. To identify copy number variations at individual exons, we compared the normalized coverage of each exon of testing patients with that of controls. Coverage data and bars/plots graph were produced using Prism 6. Exons with a normalized coverage ratio below/above 0.7/1.3 of the mean in controls were classified as heterozygously deleted/duplicated and further analyzed by MLPA [8]. A 20-bp interval was used for precise breakpoint determination.

### Sanger sequencing

Sanger sequencing was performed to confirm all detected variants. The primers Prism 3130 were designed using the online software Primer3. The amplification reaction mixture (50 μl) was subjected to denaturation at 95 °C for 2 min followed by 30 cycles at 94 °C for 1 min, annealing temperature 60 °C for 1 min, 72 °C for 1 min and by a final extension at 72 °C for 15 min. Bi-directional sequencing was performed using BigDye Terminator v3.1 Cycle Sequencing Kit, version 3.1 (Applied Biosystems, Foster, CA, USA) and analyzed on an ABI genetic analyzer (Applied Biosystems, Foster City, CA, USA).

## Results

**Fig. 1:**
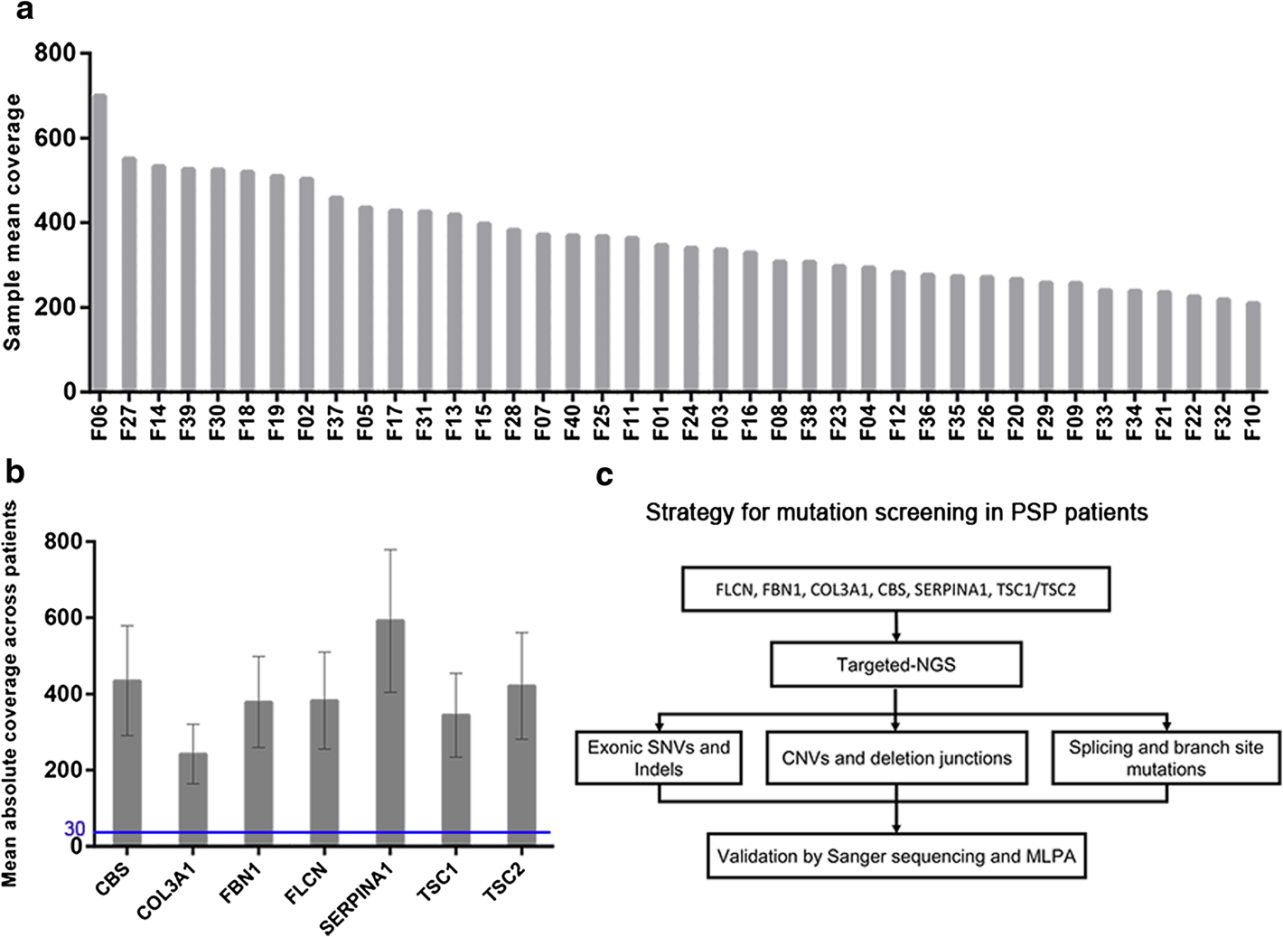
Representative Quality metrics of the targeted-NGS method and strategy for mutation screening in the targeted genes. **a** Representation of the average depth of each sample. The coverage depth was >200× for each sample. **b** Mean coverage obtained for the different genes across patients. Bar chart displaying the mean coverage of each gene in all samples. Error bars represent standard deviation. The blue line shows the lower limit of coverage appropriate to perform CNV analysis (30×). **c** Strategy for comprehensive mutation screening of the targeted genes in PSP cohort

A total of 40 samples were included in this study. Nineteen patients with 15 different types of *FLCN* pathogenic mutations previously identified by conventional Sanger sequencing and MLPA were chosen as positive controls to evaluate the performance of the target enrichment method. In addition, 21 blinded samples from genetically untested PSP patients were also examined by this method to identify the underlying mutation(s) responsible for PSP and to assess this method for its diagnostic potential. All the results were re-confirmed by conventional Sanger sequencing and MLPA. The strategy used in this study were detailed in Fig. 1c and Table 1.

### Sequencing quality metrics

The obtained sequencing data was analyzed by FastQC software, which indicated good-quality sequencing metrics, with an average 80.53 % of the sequenced bases above Q30. On average, a mean coverage of 364× was obtained per sample (ranged 210 ×–699×) (Fig. 1a). Different average coverage were obtained for the seven analyzed genes, ranging from 243× (*COL3A1*) to 592× (*SERPINA1*) (Fig. 1b). All genes were covered at more than 30× for at least 96 % of the target regions, promising the CNV analysis. The percentage of sequencing on target was 72.35 %.

### Identification of SNVs, indels and CNVs in test group: validation of the diagnostic strategy

All 15 different pathogenic sequence changes were correctly identified in the 19 positive control samples (Table 2; patients F01-F19), resulting in a diagnostic rate of 100 % for the detection of pathogenic mutations in our system. These encompassed a variety of mutations, including 2 SNVs (1 stop gain and 1 splice site mutation), 4 insertions/6 deletions ranging from 1 to 23 bases and 3 large intragenic deletions. The false-negative rate was 0 %. All samples were analyzed blind to their mutations.

**Table 2:** SNVs, Indels, and exon deletions identified by the assay in Test Group

Patient ID	Gene	RefSeq	Exon or Intron	Mutation(s) by nucleotide	Mutation(s) by amino acid	Mutation type	Conventional method of detection
F01	FLCN	NM_144997	exon11	c.1285dupC	p.H429PfsX27	Frameshift insertion, protein truncation	Sanger Sequence
F02	FLCN	NM_144997	exon11	c.1285dupC	p.H429PfsX27	Frameshift insertion, protein truncation	Sanger Sequence
F03	FLCN	NM_144997	exon14	c.1579_1580insA	p.R527QfsX75	Frameshift deletion, protein truncation	Sanger Sequence
F04	FLCN	NM_144997	exon4	c.182_186dupACAGC	p.P63TfsX69	Frameshift deletion, protein truncation	Sanger Sequence
F05	FLCN	NM_144997	exon12	c.1360dupT	p.C454LfsX2	Frameshift deletion, protein truncation	Sanger Sequence
F06	FLCN	NM_144997	exon11	c.1285delC	p.H429TfsX39	Frameshift deletion, protein truncation	Sanger Sequence
F07	FLCN	NM_144997	exon9	c.946_947delAG	p.S316YfsX73	Frameshift deletion, protein truncation	Sanger Sequence
F08	FLCN	NM_144997	exon10	c.1156_1175del	p.S386DfsX63	Frameshift deletion, protein truncation	Sanger Sequence
F09	FLCN	NM_144997	exon14	c.1648_1658del	p.L550DfsX48	Frameshift deletion, protein truncation	Sanger Sequence
F10	FLCN	NM_144997	exon6	c.469_471delTTC	p.F157del	In-frame deletion	Sanger Sequence
F11	FLCN	NM_144997	exon13	c.1522_1524delAAG	p.K508del	In-frame deletion	Sanger Sequence
F12	FLCN	NM_144997	intron4	c.250–3_268del	---	Splice site	Sanger Sequence
F13	FLCN	NM_144997	exon6	c.507G > A	p.W169X	Nonsense mutation	Sanger Sequence
F14	FLCN	NM_144997	exon6	c.507G > A	p.W169X	Nonsense mutation	Sanger Sequence
F15	FLCN	NM_144997	intron13	c.1539–1G > A	---	Splice site	Sanger Sequence
F16	FLCN	NM_144997	exon1–3	c.-504–1355_-25 + 894 del	---	Large intragenic deletion	MLPA
F17	FLCN	NM_144997	exon14	c.1539–476_1740 + 1077 del	---	Large intragenic deletion	MLPA
F18	FLCN	NM_144997	exon9–14	c.872–562_1740 + ?^a^ del	---	Large intragenic deletion	MLPA
F19	FLCN	NM_144997	exon9–14	c.872–562_1740 + ?^a^ del	---	Large intragenic deletion	MLPA

^a^The breakpoint is within the 3′ flank of the *FLCN* gene, which was not included in the panel

### Detection of exon deletions and breakpoint analysis

**Fig. 2:**
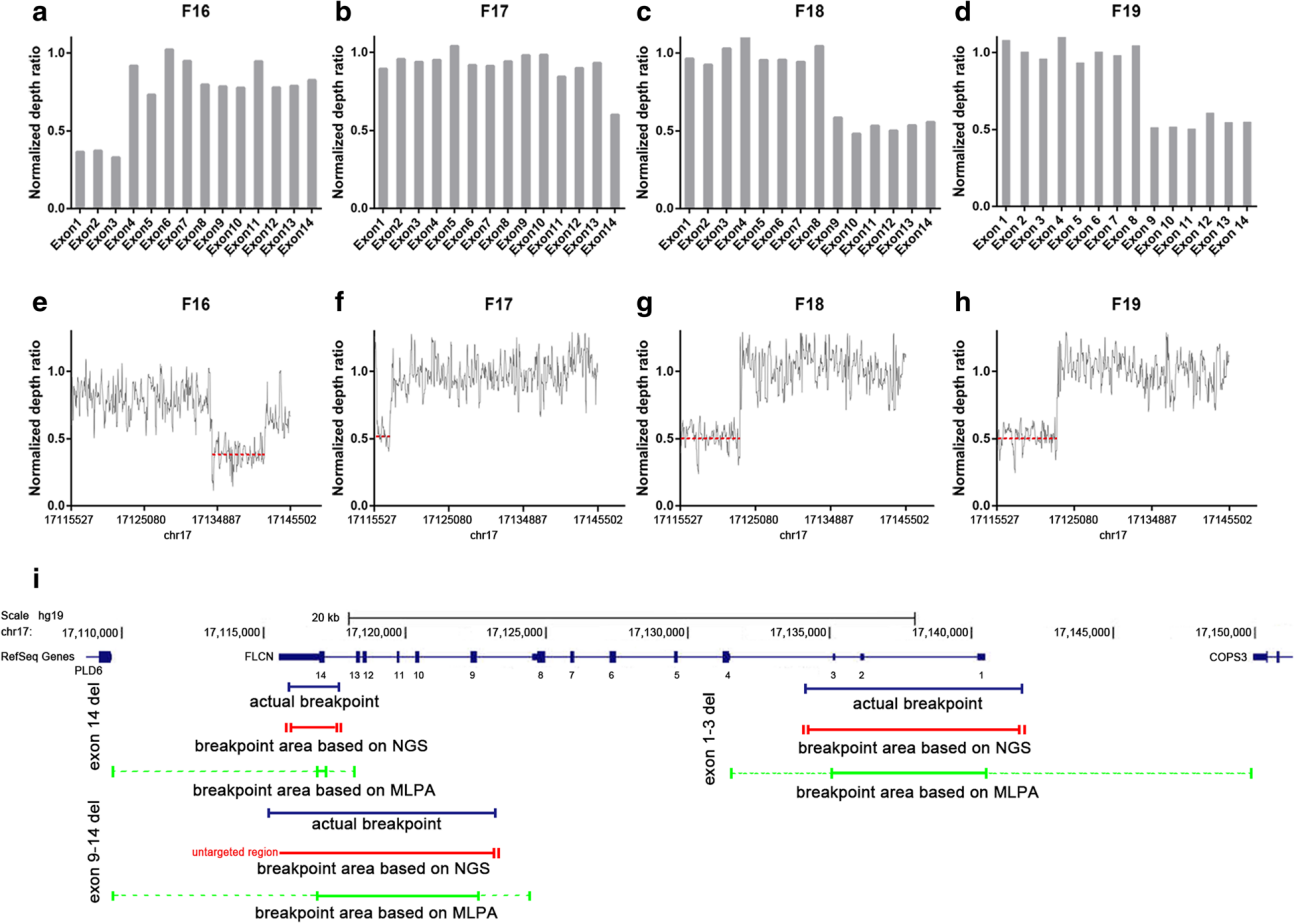
Detection of copy number variants and mapping of deletion junctions. **a**-**d** Representation of the normalized depth ratio of each exon in *FLCN* gene. Exons with a normalized coverage ratio below 0.7 were classified as heterozygously deleted. **e**-**h** Representation of the normalized depth ratio of 20-bp intervals in *FLCN* gene. The red dotted lines represent the average ratio of the deletion area. **i** The upper panel shows genomic structure of the *FLCN* gene (data from USCS database). The blue line represents actual deletion size. Deletion size of NGS-based and MLPA-based methods are represented by green line and red line, respectively. The solid line represents the definite deletion size determined by each method. Interval of vertical bars on each side of solid lines represent the range of probable deletion junctions

We observed that the coverage varied significantly between positions from the same sample, however, the samples captured with an identical protocol had very similar distribution of sequencing depth. In this context, we normalized coverage of each exon for on-target mapped bases for that sample and performed the CNV analysis using a depth-based method. This analysis allowed us to identify 4 patients with heterozygous large intragenic deletions of *FLCN* gene in our study cohort: 1 deletion in *FLCN* comprised exons 1–3 (average normalized depth ratio = 0.356). One deletion in *FLCN* comprised exon 14 (normalized depth ratio = 0.601) and 2 deletions in *FLCN* comprised exons 9–14 (average normalized depth ratio = 0.533 and 0.539, respectively) (Fig. 2a-d).

We subsequently managed to identify exact breakpoints of the deletions by calculating the normalized depth of a set of 20 bp-interval contigs covered the entire *FLCN* gene (Fig. 2e-h, Table 3). For patient F16, by using MLPA, of which probes are designed in the exons of *FLCN* gene, we detected a deletion of exons 1–3 [8]. The estimated deletion size is between 5346–18666 bp in length, while with the NGS strategy, we were able to restrict it to about 7650 bp, closed to its actual deletion size (Fig. 2i). Likewise, in patient F17 and F18/F19, we reduced the estimated size of deletion from 1645–8671 bp and 5555–13782 bp down to about 1740 bp and 6117–12017 bp, respectively (Fig. 2i).

**Table 3:** Genomic deletions and breakpoints identified by the assay

Patient ID	Gene	Genomic event	Breakpoint identified by NGS system					Breakpoint identified by amplification of the deletion junction			
			Chromosome	Start^a^	End^a^	Size (bp)	Average ratio^b^	Chromosome	Start	End	Size (bp)
F16	FLCN	Deletion exons 1–3	17	17134237	17141880	7644	0.377	17	17134286	17141828	7543
F17	FLCN	Deletion exons 14	17	17115904	17117646	1743	0.534	17	17115898	17117706	1809
F18	FLCN	Deletion exons 9–14	17	untargeted region^c^	17123085	---	0.513	17	17115206	17123002	7747
F19	FLCN	Deletion exons 9–14	17	untargeted region^c^	17123085	---	0.516	17	17115206	17123002	7747

^a^Breakpoints are defined by the external boundaries of 20 bp-intervals. Breakpoints are flanked by *Alu* repeats

^b^Average of normalized depth ratio of each 20 bp-interval

^c^The breakpoint is within the 3′ flank of the *FLCN* gene, which was not included in the panel

The breakpoints for four large intragenic deletions in *FLCN* had been identified by genomic amplification of the DNA regions across the deletion junctions, followed by Sanger sequencing. Each large deletion is flanked by *Alu* repeat sequences that mediate the mutation. Our NGS system accurately identified exon deletions and determined breakpoints on the targeted sequence within about 100 bp, largely narrowed the boundaries of the deletions, which made it much easier to design primers for genomic amplification of the deletion junction.

### Mutation analysis in blinded sample group

Our study shown there were an average of 194 variants per sample within the 214 kb targeted region in our blinded PSP cohort of PSP. After removing all SNVs/indels occurring within non-coding regions, the variations were filtered against dbSNP137 NonFlagged, 1000 genomes and ESP 6500 databases, and then submitted to PolyPhen and SIFT to evaluate possible impact of the variations on protein structure and function. Using this filtering criteria, there was 0–1 novel, predicted harmful variant remained for each sample. Of the 21 patients, we detected clearly pathogenic mutations in 3 cases (2 in *FLCN*, 1 in *FBN1*) and likely pathogenic mutations in the other 3 cases (Table 4). All of the variants were confirmed by Sanger sequencing.

**Table 4:** Mutations identified in the 21 SP patients of the blinded sample group

Patient ID	Gene	RefSeq	Exon or Intron	Mutation(s) by nucleotide	Mutation(s) by amino acid	Mutation type	Zygosity	Classification	Comments
F29	FLCN	NM_144997	exon14	c.1648_1658del	p.L550DfsX48	Frameshift deletion	Heterozygous	Definitely Pathogenic	
F37	FLCN	NM_144997	exon6	c.473delT	p.I158TfsX19	Frameshift deletion	Heterozygous	Definitely Pathogenic	
F32	FBN1	NM_000138	exon51	c.6169C > T	p.R2057X	Stopgain SNV	Heterozygous	Definitely Pathogenic	Not reported in any database
F27	FBN1	NM_000138	exon19	c.2269G > C	p.D757H	Missense SNV	Heterozygous	Likely Pathogenic	Situates in the EGF-like calcium-binding domain of FBN1; highly conserved; predicted damaging
F33	TSC1	NM_000368	exon15	c.1631G > A	p.G544E	Missense SNV	Heterozygous	Likely Pathogenic	Highly conserved; predicted damaging
F34	FLCN	NM_144997	intron10	c.1177–21G > A	---	Splicing	Heterozygous	Likely Pathogenic	Branch Site mutation

**Fig. 3:**
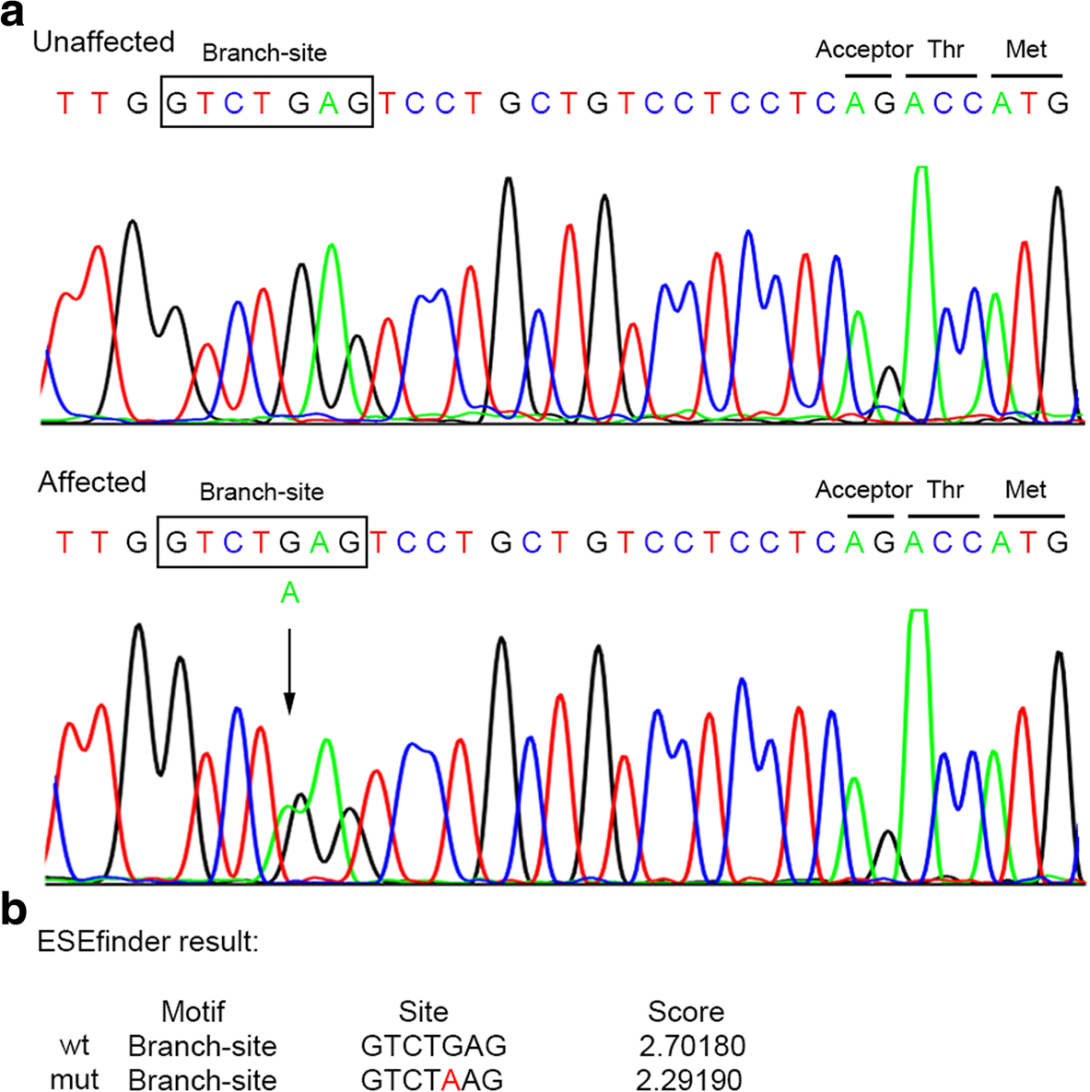
Sanger sequencing and ESEfinder results of the branch-site mutation. **a** Sequencing chromatograms show a heterozygous mutation c.1177–21G > A in *FLCN* gene. The arrow indicates the position of the nucleotide mutation and the boxed nucleotides indicate the branch site predicted by ESEfinder. **b** ESEfinder results for the c.1177–21G > A substitution and for the wild type of the branch-site sequence

Two small frameshift deletions were identified in *FLCN* gene in patients F29 and F37, both resulting in a truncated protein of FLCN. And one of them, a c.1648_1658 deletion in exon 14 of the *FLCN* gene had been detected in patient F09. A nonsense mutation were detected in *FBN1* gene in patient F32, it was a C to T transition (c.6169 C > T) in exon 51, predicting an early stop codon at position 2057 (p.Arg2057Ter) which causes a premature termination of the protein. This mutation has not been recorded in any database including *FBN1* mutation database [41]. In this unscreened cohort of PSP patients, we also identified two missense mutations predicted to be damaging by SIFT and Polyphen2. One of them was a c.2269G > C transversion in exon 19 of the *FBN1* gene, which converted the codon GAT for Aspartic at position 757 to CAT, a codon for Histidine. This variant is predicted by ScanProsite to situate in the EGF-like calcium-binding domain of FBN1 [42]. The other was a c.1631G > A transition in exon 15 of the *TSC1* gene, which was predicted to substitute a polar uncharged glycine residue for a negatively charged glutamic residue (p.Gly544Glu), presenting a distinct change in amino acid properties. We also detected a G to A point mutation in intron 10 (c.1177–21G > A) of *FLCN* gene (Fig. 3a). This variant located within the branch-site sequence, by using the ESEfinder, we obtained a matrix score for the c.1177–21G > A substitution (2.2919) of the branch-site sequence, relatively less than a score for the wild type (2.7018) (Fig. 3b), indicating that it may disrupt the branch-site in this gene. However, because of unavailability of the patient’s tissue, we were unable to verify it at transcript level.

In general, NGS method not only successfully identified missense, nonsense, splice site, intronic branch site, small indel, and large deletion changes in positive and blinded samples, but also determined approximate deletion junction within about 100 bp deviation by using a depth-based method.

## Discussion

In this study, to establish a genetic diagnostic method for PSP, we evaluated the performance of targeted enrichment next-generation sequencing for the molecular diagnosis of PSP patients. We enriched 7 genes which are known to cause PSP using our self-designed Haloplex panel (Table 1) and sequenced them on Miseq platform. In test group, our target-enrichment NGS method successfully identified all 19 pathogenic mutations including SNVs, Indels and large intragenic mutations previously detected by Sanger sequencing and MLPA. No false negative result in test group was obtained. Moreover, we also detected the breakpoints of the deletions within 100 bp using a depth-based bioinformatics method. In blinded sample group, we detected 3 definitely pathogenic mutations including a novel *FBN1* mutation and 3 likely pathogenic mutations including an intronic branch-site mutation. Therefore, we conclude that our system has a more extensive performance than Sanger sequencing and MLPA.

As BHD patients often receive medical attention for their pneumothorax onset in the hospital, genetic screening in PSP patients will help early diagnose of BHD syndrome, thus lead to early recognition and treatment of renal cell carcinoma. However, for screening, Sanger sequencing and MLPA are relatively low throughput, inefficient and expensive. Our targeted next-generation sequencing method successfully and efficiently identifies full spectrum of *FLCN* gene mutations in a single experiment.

By using a depth-based bioinformatics method, we also narrowed the range of breakpoints to a great extent, but we did not identify the precise breakpoints of the deletions, as it is reported that all *FLCN* intragenic deletions are *Alu* repeats-mediated [8], yet the low sensitivity in detecting CNVs in repeat regions is not completely solved [43], and Sanger sequencing is still the gold standard for breakpoint determination. Our NGS approach thus obviates the need for separate testing for genomic deletions/duplications after Sanger sequencing, and can also largely minimize the boundaries of the deletions, which will make it much easier to design primers for long-range PCR amplification. Our limitation is that one of the breakpoint of exon 9–14 deletion was not recovered since our design did not include the 3′ flank of *FLCN* gene. To optimize our method, the 5′ flank and 3′ flank sequences of tested genes should be included.

In the blinded PSP cohort, two out of twenty-one patients (9.5 %) were identified to carry pathogenic mutations in *FLCN* gene. This is in concordance with the previous report that up to 10 % of PSP patients are carrying *FLCN* mutations [7, 8]. A novel nonsense mutation in *FBN1* (c.6169C > T; p.Arg2057Ter) was found in a male PSP patient. It was classified as definitely pathogenic mutation and was not recorded in any database. Our finding expand the spectrum of mutations in Marfan syndrome. Interestingly, a novel intronic variant in *FLCN* (c.1177–21G > A) was also detected by this method, bringing forward the need of raising awareness of intronic variant in pathogenesis, which is often avoided by traditional Sanger sequence.

Among the targeted genes, we also identified three mutations in *FBN1* and *TSC1* gene in PSP patients in addition to *FLCN* gene, but no predicted harmful variant was detected in *TSC2*, *CBS*, *COL3A1* and *SERPINA1* gene. Patients with mutations in these genes often express as syndromes and can be easily diagnosed. Isolated spontaneous pneumothorax patients with mutations in these genes are rare [44]. Nevertheless, pulmonologists should consider these syndromes and sequence these genes when treating patients for spontaneous pneumothorax, since early detection improves the prognosis significantly.

Moreover, a cost analysis of NGS approach showed significant savings. The cost with conventional Sanger sequencing is about $1760 per sample for 220 PCR and sequencing reactions, and the cost with 7 MLPA reactions is about $110 per sample. While a total cost of Haloplex target enrichment sequencing is about $400. With our NGS-based assay, a 78 % of cost savings per sample could be achieved in PSP diagnostic procedure.

## Conclusions

This is the first study to evaluate the performance of NGS in the detection of exonic and intronic SNVs, Indels and large intragenic deletions, and to develop a cost- and time-effective screening system for the molecular diagnosis of BHDS in PSP cohort. In conclusion, NGS based Haloplex target enrichment technology is a rapid, high-throughput and cost-effective screening strategy for the molecular diagnosis of BHD in PSP patients, as it can replace Sanger sequencing and MLPA by simultaneously detecting exonic and intronic SNVs, small indels, large intragenic deletions and determining deletion junctions in PSP-related genes.

## Abbreviations

BHD: birt-hogg-dube syndrome
CNV: copy number variations
MLPA: multiple ligation-dependent probe amplification
NGS: next generation sequencing
PSP: primary spontaneous pneumothorax
RCC: renal cell carcinoma

## References

[1] Abolnik IZ, Lossos IS, Zlotogora J, Brauer R (1991). On the inheritance of primary spontaneous pneumothorax. Am J Med Genet.

[2] Yellin A, Shiner RJ, Lieberman Y (1991). Familial multiple bilateral pneumothorax associated with Marfan syndrome. Chest.

[3] Ishiguro T, Takayanagi N, Kawabata Y, Matsushima H, Yoshii Y, Harasawa K, Yamaguchi S, Yoneda K, Miyahara Y, Kagiyama N, Tokunaga D, Aoki F, Saito H, Kurashima K, Ubukata M, Yanagisawa T, Sugita Y, Okita H, Hatamochi A (2009). Ehlers-Danlos syndrome with recurrent spontaneous pneumothoraces and cavitary lesion on chest X-ray as the initial complications. Intern Med.

[4] Bass HN, LaGrave D, Mardach R, Cederbaum SD, Fuster CD, Chetty M (1997). Spontaneous pneumothorax in association with pyridoxine-responsive homocystinuria. J Inherit Metab Dis.

[5] Serapinas D, Obrikyte V, Vaicius D, Balciuviene R, Valavicius A, Sakalauskas R (2014). Alpha-1 antitrypsin deficiency and spontaneous pneumothorax: possible causal relationship. Pneumologia.

[6] Valentin-Mendoza S, Nieves-Nieves J, Fernandez-Medero R, Fernandez-Gonzales R, Adorno-Fontanez J, Adorno-Fontanez E (2013). Pulmonary lymphangioleiomyomatosis: literature update. Bol Asoc Med P R.

[7] Ren HZ, Zhu CC, Yang C, Chen SL, Xie J, Hou YY, Xu ZF, Wang DJ, Mu DK, Ma DH, Wang Y, Ye MH, Ye ZR, Chen BF, Wang CG, Lin J, Qiao D, Yi L (2008). Mutation analysis of the FLCN gene in Chinese patients with sporadic and familial isolated primary spontaneous pneumothorax. Clin Genet.

[8] Ding Y, Zhu C, Zou W, Ma D, Min H, Chen B, Ye M, Pan Y, Cao L, Wan Y, Zhang W, Meng L, Mei Y, Yang C, Chen S, Gao Q, Yi L (2015). FLCN intragenic deletions in Chinese familial primary spontaneous pneumothorax. Am J Med Genet A.

[9] Schmidt LS, Warren MB, Nickerson ML, Weirich G, Matrosova V, Toro JR, Turner ML, Duray P, Merino M, Hewitt S, Pavlovich CP, Glenn G, Greenberg CR, Linehan WM, Zbar B (2001). Birt-Hogg-Dube syndrome, a genodermatosis associated with spontaneous pneumothorax and kidney neoplasia, maps to chromosome 17p11.2. Am J Hum Genet.

[10] Nickerson ML, Warren MB, Toro JR, Matrosova V, Glenn G, Turner ML, Duray P, Merino M, Choyke P, Pavlovich CP, Sharma N, Walther M, Munroe D, Hill R, Maher E, Greenberg C, Lerman MI, Linehan WM, Zbar B, Schmidt LS (2002). Mutations in a novel gene lead to kidney tumors, lung wall defects, and benign tumors of the hair follicle in patients with the Birt-Hogg-Dube syndrome. Cancer Cell.

[11] Johannesma PC, Reinhard R, Kon Y, Sriram JD, Smit HJ, van Moorselaar RJ, Menko FH, Postmus PE (2015). Prevalence of Birt-Hogg-Dube syndrome in patients with apparently primary spontaneous pneumothorax. Eur Respir J.

[12] Birt AR, Hogg GR, Dube WJ (1977). Hereditary multiple fibrofolliculomas with trichodiscomas and acrochordons. Arch Dermatol.

[13] Roth JS, Rabinowitz AD, Benson M, Grossman ME (1993). Bilateral renal cell carcinoma in the Birt-Hogg-Dube syndrome. J Am Acad Dermatol.

[14] Toro JR, Glenn G, Duray P, Darling T, Weirich G, Zbar B, Linehan M, Turner ML (1999). Birt-Hogg-Dube syndrome: a novel marker of kidney neoplasia. Arch Dermatol.

[15] Toro JR, Pautler SE, Stewart L, Glenn GM, Weinreich M, Toure O, Wei MH, Schmidt LS, Davis L, Zbar B, Choyke P, Steinberg SM, Nguyen DM, Linehan WM (2007). Lung cysts, spontaneous pneumothorax, and genetic associations in 89 families with Birt-Hogg-Dube syndrome. Am J Respir Crit Care Med.

[16] Maffe A, Toschi B, Circo G, Giachino D, Giglio S, Rizzo A, Carloni A, Poletti V, Tomassetti S, Ginardi C, Ungari S, Genuardi M (2011). Constitutional FLCN mutations in patients with suspected Birt-Hogg-Dube syndrome ascertained for non-cutaneous manifestations. Clin Genet.

[17] Menko FH, van Steensel MA, Giraud S, Friis-Hansen L, Richard S, Ungari S, Nordenskjold M, Hansen TV, Solly J, Maher ER (2009). Birt-Hogg-Dube syndrome: diagnosis and management. Lancet Oncol.

[18] Frohlich BA, Zeitz C, Matyas G, Alkadhi H, Tuor C, Berger W, Russi EW (2008). Novel mutations in the folliculin gene associated with spontaneous pneumothorax. Eur Respir J.

[19] Graham RB, Nolasco M, Peterlin B, Garcia CK (2005). Nonsense mutations in folliculin presenting as isolated familial spontaneous pneumothorax in adults. Am J Respir Crit Care Med.

[20] Gunji Y, Akiyoshi T, Sato T, Kurihara M, Tominaga S, Takahashi K, Seyama K (2007). Mutations of the Birt Hogg Dube gene in patients with multiple lung cysts and recurrent pneumothorax. J Med Genet.

[21] Kunogi M, Kurihara M, Ikegami TS, Kobayashi T, Shindo N, Kumasaka T, Gunji Y, Kikkawa M, Iwakami S, Hino O, Takahashi K, Seyama K (2010). Clinical and genetic spectrum of Birt-Hogg-Dube syndrome patients in whom pneumothorax and/or multiple lung cysts are the presenting feature. J Med Genet.

[22] Painter JN, Tapanainen H, Somer M, Tukiainen P, Aittomaki K (2005). A 4-bp deletion in the Birt-Hogg-Dube gene (FLCN) causes dominantly inherited spontaneous pneumothorax. Am J Hum Genet.

[23] Yang CY, Wang HC, Chen JS, Yu CJ (2013). Isolated familial pneumothorax in a Taiwanese family with Birt-Hogg-Dube syndrome. J Postgrad Med.

[24] Houweling AC, Gijezen LM, Jonker MA, van Doorn MB, Oldenburg RA, van Spaendonck-Zwarts KY, Leter EM, van Os TA, van Grieken NC, Jaspars EH, de Jong MM, Bongers EM, Johannesma PC, Postmus PE, van Moorselaar RJ, van Waesberghe JH, Starink TM, van Steensel MA, Gille JJ, Menko FH (2011). Renal cancer and pneumothorax risk in Birt-Hogg-Dube syndrome; an analysis of 115 FLCN mutation carriers from 35 BHD families. Br J Cancer.

[25] Zbar B, Alvord WG, Glenn G, Turner M, Pavlovich CP, Schmidt L, Walther M, Choyke P, Weirich G, Hewitt SM, Duray P, Gabril F, Greenberg C, Merino MJ, Toro J, Linehan WM (2002). Risk of renal and colonic neoplasms and spontaneous pneumothorax in the Birt-Hogg-Dube syndrome. Cancer Epidemiol Biomarkers Prev.

[26] Wei MH, Blake PW, Shevchenko J, Toro JR (2009). The folliculin mutation database: an online database of mutations associated with Birt-Hogg-Dube syndrome. Hum Mutat.

[27] Lim DH, Rehal PK, Nahorski MS, Macdonald F, Claessens T, Van Geel M, Gijezen L, Gille JJ, Giraud S, Richard S, van Steensel M, Menko FH, Maher ER (2010). A new locus-specific database (LSDB) for mutations in the folliculin (FLCN) gene. Hum Mutat.

[28] Benhammou JN, Vocke CD, Santani A, Schmidt LS, Baba M, Seyama K, Wu X, Korolevich S, Nathanson KL, Stolle CA, Linehan WM (2011). Identification of intragenic deletions and duplication in the FLCN gene in Birt-Hogg-Dube syndrome. Genes Chromosomes Cancer.

[29] Walsh T, Lee MK, Casadei S, Thornton AM, Stray SM, Pennil C, Nord AS, Mandell JB, Swisher EM, King MC (2010). Detection of inherited mutations for breast and ovarian cancer using genomic capture and massively parallel sequencing. Proc Natl Acad Sci U S A.

[30] Valencia CA, Rhodenizer D, Bhide S, Chin E, Littlejohn MR, Keong LM, Rutkowski A, Bonnemann C, Hegde M (2012). Assessment of target enrichment platforms using massively parallel sequencing for the mutation detection for congenital muscular dystrophy. J Mol Diagn.

[31] Sperandeo MP, Panico M, Pepe A, Candito M, de Franchis R, Kraus JP, Andria G, Sebastio G (1995). Molecular analysis of patients affected by homocystinuria due to cystathionine beta-synthase deficiency: report of a new mutation in exon 8 and a deletion in intron 11. J Inherit Metab Dis.

[32] Superti-Furga A, Gugler E, Gitzelmann R, Steinmann B (1988). Ehlers-Danlos syndrome type IV: a multi-exon deletion in one of the two COL3A1 alleles affecting structure, stability, and processing of type III procollagen. J Biol Chem.

[33] Jones AC, Daniells CE, Snell RG, Tachataki M, Idziaszczyk SA, Krawczak M, Sampson JR, Cheadle JP (1997). Molecular genetic and phenotypic analysis reveals differences between TSC1 and TSC2 associated familial and sporadic tuberous sclerosis. Hum Mol Genet.

[34] Blyth M, Foulds N, Turner C, Bunyan D (2008). Severe Marfan syndrome due to FBN1 exon deletions. Am J Med Genet.

[35] Wang K, Li M, Hakonarson H (2010). ANNOVAR: functional annotation of genetic variants from high-throughput sequencing data. Nucleic Acids Res.

[36] Adzhubei IA, Schmidt S, Peshkin L, Ramensky VE, Gerasimova A, Bork P, Kondrashov AS, Sunyaev SR (2010). A method and server for predicting damaging missense mutations. Nat Methods.

[37] Kumar P, Henikoff S, Ng PC (2009). Predicting the effects of coding non-synonymous variants on protein function using the SIFT algorithm. Nat Protoc.

[38] Cartegni L, Wang J, Zhu Z, Zhang MQ, Krainer AR (2003). ESEfinder: a web resource to identify exonic splicing enhancers. Nucleic Acids Res.

[39] McKenna A, Hanna M, Banks E, Sivachenko A, Cibulskis K, Kernytsky A, Garimella K, Altshuler D, Gabriel S, Daly M, DePristo MA (2010). The genome analysis toolkit: a MapReduce framework for analyzing next-generation DNA sequencing data. Genome Res.

[40] DePristo MA, Banks E, Poplin R, Garimella KV, Maguire JR, Hartl C, Philippakis AA, del Angel G, Rivas MA, Hanna M, McKenna A, Fennell TJ, Kernytsky AM, Sivachenko AY, Cibulskis K, Gabriel SB, Altshuler D, Daly MJ (2011). A framework for variation discovery and genotyping using next-generation DNA sequencing data. Nat Genet.

[41] Collod-Beroud G, Le Bourdelles S, Ades L, Ala-Kokko L, Booms P, Boxer M, Child A, Comeglio P, De Paepe A, Hyland JC, Holman K, Kaitila I, Loeys B, Matyas G, Nuytinck L, Peltonen L, Rantamaki T, Robinson P, Steinmann B, Junien C, Beroud C, Boileau C (2003). Update of the UMD-FBN1 mutation database and creation of an FBN1 polymorphism database. Hum Mutat.

[42] de Castro E, Sigrist CJ, Gattiker A, Bulliard V, Langendijk-Genevaux PS, Gasteiger E, Bairoch A, Hulo N. ScanProsite: detection of PROSITE signature matches and ProRule-associated functional and structural residues in proteins. Nucleic Acids Res. 2006;34(Web Server issue):365–5. doi:10.1093/nar/gkl124. 10.1093/nar/gkl124 PMC1538847 16845026

[43] Teo SM, Pawitan Y, Ku CS, Chia KS, Salim A (2012). Statistical challenges associated with detecting copy number variations with next-generation sequencing. Bioinformatics.

[44] Chiu HT, Garcia CK (2006). Familial spontaneous pneumothorax. Curr Opin Pulm Med.

